# Opinionated Views on Grassland Restoration Programs on the Qinghai-Tibetan Plateau

**DOI:** 10.3389/fpls.2022.861200

**Published:** 2022-04-26

**Authors:** Ting Hua, Wenwu Zhao, Paulo Pereira

**Affiliations:** ^1^State Key Laboratory of Earth Surface Processes and Resource Ecology, Faculty of Geographical Science, Beijing Normal University, Beijing, China; ^2^Institute of Land Surface System and Sustainable Development, Faculty of Geographical Science, Beijing Normal University, Beijing, China; ^3^Environmental Management Center, Mykolas Romeris University, Vilnius, Lithuania

**Keywords:** grassland degradation, ecological restoration, protected areas, grassland management, grazing, Qinghai-Tibetan plateau

## Introduction

The Qinghai-Tibet Plateau (QTP), also called the Third Pole, is the largest high-elevation region with an area of 2.5 million km^2^ and an average elevation of about 4000 m, mainly including Tibet, Qinghai, and the north-west of Sichuan. Alpine grasslands, such as alpine meadows, alpine steppes, and alpine desert steppes, are the dominant biomes on the QTP. It is also a key source for several major Asian rivers and supplies essential materials and ecosystem services (e.g., water, food) to over a billion people downstream (Immerzeel et al., 2020). Grassland is the most common ecosystem, occupying more than 60% of the QTP (Dong and Sherman, 2015), and plays a critical role in regional ecological security and economic development (Sun et al., 2021). However, climate change and anthropogenic activities, such as overgrazing, resulted in widespread and intensive grassland degradation (Dong et al., 2020), affecting regional sustainability. The Chinese government has supported an integrated portfolio of large-scale grassland ecological restoration programs since 2000. Among them are ecological engineering, protected areas (PAs), and other forms to support local communities through ecological compensation and ecological migration (i.e., transferring scattered residents from ecologically vulnerable areas and allocating them in towns to reduce human pressure and protect the degraded ecosystems). These actions improved regional environmental status and promoted multiple Sustainable Development Goals (SDGs), while several adverse consequences also occurred, including biodiversity threat and water resources crisis. The Chinese government is planning a new round of ecological restoration on the QTP. For instance, the national master plan for major projects to protect and restore the ecosystem (2021–2035) has been released, and the ecological issues of QTP are on top of priorities. Therefore, it is necessary to identify some key characteristics and potential gaps of existing ecological programs. Here, we reviewed these ecological protection and construction schemes conducted in QTP and proposed potential implications for grassland ecosystem restoration. This synthesis aimed to help and improve future grassland restoration programs or policies on QTP and provide guidance and experiences for other regions that seek to tackle similar issues.

## Causes of Grassland Degradation

Internal and external factors cause the grassland degradation on the QTP. As an internal factor, the Alpine grassland ecosystem has a slow energy flow and material circulation rate (e.g., the carbon cycle) (Shang and Long, 2007). In this ecosystem, the low organic matter decomposition results in the high accumulation of undecomposed organic material on the soil surface. Also, soil nutrients in Alpine grasslands are low, reducing grassland regeneration capacity (Shang and Long, 2007; Liu et al., 2018). When the turf layer is removed, the underlying layer and the soil is bare, thus restricting the resilience of the grassland ecosystem (Cao and Long, 2009). As external factors, the increasing human population, livestock, agricultural expansion, mining, and urbanization, affect dramatically grassland ecosystems (Shang and Long, 2007; Wu et al., 2018). It is estimated that non-climatic factors accounted for 66.1% of grassland change on the QTP (Pan et al., 2017). However, climate change also exerts an important influence. Vegetation growth in alpine regions is susceptible to changes in precipitation and temperature (Xiong et al., 2019). The warmer temperature might lead to earlier spring phenology, long growth periods, and a high photosynthesis rate, enhancing carbon assimilation and biomass production (Xiong et al., 2016). However, temperature increase also causes frozen soil melting, destroying the structure of vegetation root systems and hindering growth (Xiong et al., 2016). Therefore, the causes of grassland degradation on the QTP are complex and diverse.

## Effects of Grassland Degradation

**Figure 1:**
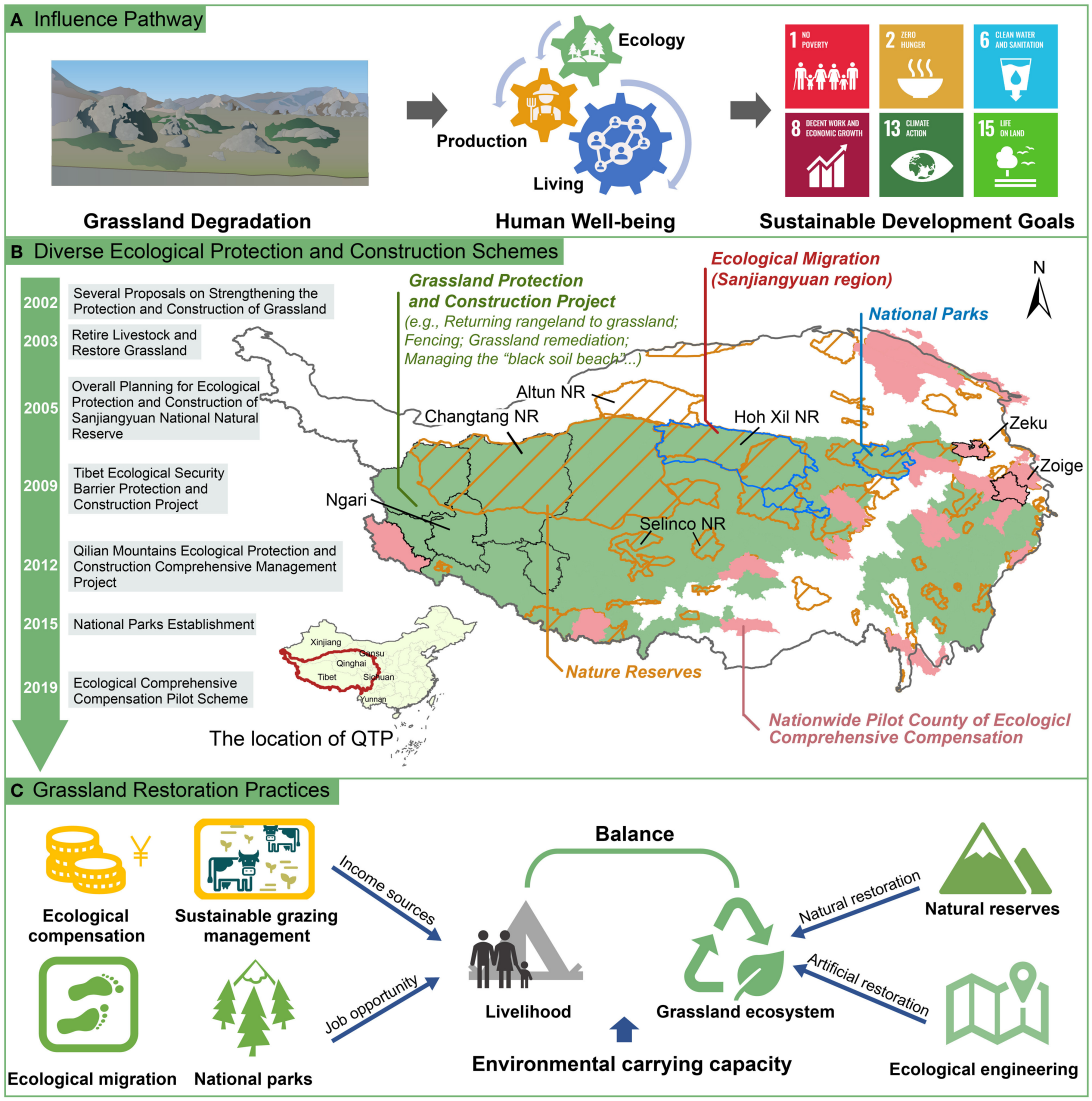
Effects of grassland degradation and its restoration practices. **(A)** influence pathway of grassland degradation on human well-being and SDGs, **(B)** distribution and timeline of grassland ecological protection and construction schemes, and **(C)** potential implications for grassland ecosystem restoration. NR means nature reserves.

It is widely acknowledged that the QTP's grassland ecosystems have been severely degraded over the last half-century (Dong and Sherman, 2015; Liu et al., 2018). As shown in Figure 1A, grassland degradation negatively impacts ecology, production, and living dimensions. The loss of vegetation cover resulted in direct or indirect biodiversity degradation and the supply of ecosystem services, such as water conservation or carbon sequestration. A literature review carried out by Liu et al. (2018) in QTP found that degraded grasslands have on average 42% lower carbon stocks than non-degraded grasslands. This degradation may affect the alpine grassland ecosystems from a carbon fixing source to a carbon emission one (Ma et al., 2018). Also, due to vegetation loss, turf peeling, and poisonous weeds, grassland degradation greatly reduces plant diversity, species richness, and soil fauna richness (Li et al., 2015). Besides, grassland degradation exacerbates nutrient losses by erosion and leaching, increasing surface and groundwater pollution (Vitousek et al., 2010; Zhang et al., 2013; Liu et al., 2018). Also, nutrient losses limit forage production (Guo et al., 2019). This negatively affects livestock production and reproduction, harming animal husbandry development and herders' livelihoods (Dong et al., 2020). In this context, grassland degradation can have detrimental impacts on several SDGs, such as SDG1 (No Poverty), SDG2 (Zero Hunger), SDG6 (Clean Water and Sanitation), SDG8 (Decent Work and Economic growth), SDG13 (Climate Action), and SDG15 (Life on Land).

## Diverse Ecological Protection and Construction Schemes

Since 2000, the Chinese government and some provincial governments, including Qinghai and Tibet, have implemented a series of ecological protection and restoration programs to revert grassland degradation and maintain socio-ecosystem sustainability (Table 1, Figure 1B). It includes “Several Proposals on Strengthening the Protection and Construction of Grassland” sponsored by China State Council (2002), Retire Livestock and Restore Grassland (2003), Overall planning for ecological protection and construction of Sanjiangyuan National Natural Reserve (2005), Tibet ecological security barrier protection and construction project (2009), Qilian Mountains ecological protection and construction comprehensive management project (2012), the establishment of National Parks (2015) and ecological comprehensive compensation pilot scheme (2019). These programs aimed to improve directly or indirectly vulnerable grassland ecosystems through natural/artificial restoration or to alleviate human pressure (e.g., grazing).

**Table 1:** Diverse ecological protection and construction schemes.

**Year**	**Program name**	**Targeted area**	**Objectives or consequences**	**Division**
2002	Several proposals on strengthening the protection and construction of grassland	Major grassland and pastoral areas	Improve the ecological environment of grassland and promote a virtuous cycle for grassland ecology	State Council of the People's Republic of China
2003	Retire livestock and restore grassland	Major grassland and pastoral areas	Restore and construct grassland ecosystem by strictly controlling the amount of livestock carried, fences and constructing artificial forage grass foundations	State Council of the People's Republic of China
2005	Overall planning for ecological protection and construction of Sanjiangyuan National Natural Reserve	Sanjiangyuan, Qinghai Province	The vegetation coverage was increased, the capacity of soil and water conservation was enhanced, and grazing intensity was reduced	State Council of the People's Republic of China
2009	Tibet ecological security barrier protection and construction project	Tibet Autonomous Region	Improve the degraded grassland and conduct rodent pest management, and alleviate the contradiction between grass and livestock	National Development and Reform Commission of the People's Republic of China
2012	Qilian Mountains ecological protection and construction comprehensive management project	Qinghai Province, Gansu Province	Cumulatively invested 3.5 billion CNY. Protect and restore the ecosystems in Qilian Mountain area and greatly improve the production and living conditions of farmers and herders.	National Development and Reform Commission of the People's Republic of China
2015	National parks establishment	PAs in China	Improve the efficiency of PAs management, reduce human disturbance and restore grassland ecosystem. Sanjiangyuan National Park was established	State Council of the People's Republic of China
2019	Ecological comprehensive compensation pilot scheme	10 provinces in China	Establish an ecological compensation system, and increase the efficiency of ecological compensation funds	National Development and Reform Commission of the People's Republic of China

According to the above-mentioned ecological programs, grassland protection and construction projects covered nearly half of the QTP counties. These projects intended to reverse grassland degradation by returning rangeland to grassland, fencing, grassland remediation, and managing the “black soil beach” (i.e., a severely degraded alpine meadow on the QTP), to mention some. Following the adoption of these programs, grassland ecosystems progressively recovered from intense grazing pressure. The human-induced degradation was reduced, particularly in the Sanjiangyuan region (Cai et al., 2015; Xu et al., 2016). However, the projected positive impact was not observed in all regions. This could be attributed to the restoration approach chosen and the biogeographical characteristics of these areas. For instance, in the “black soil beach” programs, weeds removal was one of the primary restoration measures. This reduces vegetation cover quickly, but it is beneficial to the long-term development of native species. The delayed effectiveness of “black soil beach” programs in some regions (e.g., Zeku) confirmed it (Cai et al., 2015). Besides, the main focus of grazing prohibition and reduced grazing was to reduce the intensity through fencing, allowing grasslands to recover naturally. The biogeographical location also affected the programs' effectiveness since climatic factors are closely associated with biogeographical areas and main vegetation growth drivers (Shen et al., 2015; Yao et al., 2018). Climatic factors, including temperature and precipitation, can have synergies or trade-offs with the established ecological programs, affecting their success. For instance, ecological engineering interventions have limited effectiveness in the alpine desert steppe, where precipitation is low (e.g., Ngari Prefecture). However, they can be effective in regions with abundant rainfall (e.g., Zoige area) (Sun et al., 2021).

The adverse effect of ecological restoration policies on water resources, soil, and biodiversity was also reported (Sun et al., 2021; Xiao et al., 2021). For instance, vegetation cover increases evapotranspiration. Xiao et al. (2021) found that the potential evapotranspiration in Yarlung Zangbo River Basin can reach 650 mm yr^−1^, exceeding the average annual precipitation (360 mm). The drought caused by this unsustainable green development may jeopardize the restoration program's outcomes (Cao and Zhang, 2015). Also, long-term fencing for grazing exclusion may decrease soil fertility (Wu et al., 2021). For example, the Zoige alpine meadows experiment showed that fencing significantly decreased 15% of soil organic carbon and total nitrogen in the 0–20 cm soil layer (Wu et al., 2021). Dense fencing networks restricted wildlife movement and caused habitat fragmentation, creating challenges to biodiversity conservation on the QTP (Sun et al., 2020). The cases mentioned above highlighted the urgency to comprehensively weigh the long-term benefits and trade-offs of ecological restoration.

The study areas include a dense network of PAs. National nature reserves (NRs) spanning over 30% of QTP. The establishment of NRs is vital to decrease human activities and control grazing, which is essential for grassland recovery (Li et al., 2018). It is estimated that over 70% of PAs have a higher vegetation growth than in non-protected land (Zhang et al., 2016). Also, these NRs can have a favourable spillover effect on neighbourhood areas up to 20 kilometres away (Shen et al., 2021). It is an encouraging hint that PAs can play a remarkably effective role in enhancing grassland ecosystems and promoting nearby areas. However, except for Sanjiangyuan Reserve, where the restoration aimed to safeguard the alpine grasslands, other large-scale national NRs (e.g., Altun, Hoh Xil, and Changtang NRs) are primarily concerned with ungulates (Su et al., 2019). The importance of PAs in improving vegetation has not been adequately recognized. The Tibetan-inhabited settlements are typically located in the areas adjacent to NRs. Some major roads or railways fragmented the NRs, such as Sanjiangyuan, Hoh Xil, and Selinco, and may affect NRs biodiversity and protection capacity (Pack et al., 2016; Hua et al., 2022). The government strives to manage the PAs system with national parks as the main body, aimed to optimize their effectiveness, the fragmented management, and the questionable PAs distribution (Xu et al., 2019). For instance, in Sanjiangyuan National Park, one of the responsibilities was to explore the mode to balance the relationships between a local farmer and herders' activities and the vitality of the ecosystems. The national parks system will be expanded to include more areas in QTP.

In QTP, grazing is the main human pressure and the primary source of livelihood in alpine grasslands (Song et al., 2009; Li et al., 2013). To control grazing intensity, the government initiated ecological compensation and ecological migration measures as a complement to ecological projects and PAs (Figure 1). For instance, at the end of 2007 (driven by the ecological migration programs in Sanjiangyuan), over 60 000 people were moved from the core and buffer area of Sanjiangyuan NR and started livelihoods in cities and towns (Wang et al., 2010). The government offered the subsistence allowance and job opportunities to cover herders' economic losses resulting from protection measures. These actions increased grassland coverage and biodiversity after ecological migration (Jiang and Dai, 2009). However, as previous studies highlighted, if the payments are not enough for subsistence, part herders may expand grazing, degrading grassland (Bennett, 2008; Zhen et al., 2014). For this reason, in 2011–2015, more than half of the counties in Tibet enacted subsidies and rewards for grassland ecological protection. In 2019, twenty-three counties in QTP were included in a nationwide pilot plan for comprehensive ecological compensation. These approaches are projected to improve the compensation funds' efficiency while also strengthening the endogenous drive of diverse stakeholders.

## Potential Implications for Grassland Ecosystem Restoration

The current ecological protection and construction schemes reduced grassland degradation. These measures can be split into two categories 1) natural restoration (e.g., NRs) and 2) artificial restoration (e.g., returning rangeland to grassland). Natural restoration reduces external disturbance, using ecosystem resilience and natural succession to improve the ecosystems. Artificial restoration aims to recover degraded ecosystems under varying degrees of human impact. The implementation of grassland protection and restoration should be carried out according to local specificities such as biogeography patterns, traditional human activities, the efficiency of different measures implemented, and the socio-ecological cost. Due to climate change and human pressure, the ecosystems have varying degrees of sensitivity, resilience, and exposure to external disturbances (Li et al., 2018). Some ecological projects, such as grazing exclusion with fences, in an alpine meadow with high precipitation (e.g., Zoige area), maybe more effective than if applied in the alpine desert steppe due to the low precipitation (Sun et al., 2021). In addition, future scenarios (e.g., CMIP6 model) can be used to assess the impact of future climate change on grassland ecosystems and the services provided, as highlighted by Li et al. (2018) and Hua et al. (2021). Some precautionary and proactive interventions are required to deal with the high-sensitivity places where grasslands are severely impacted by climate change and human activities, such as artificial grassland restoration projects. In addition, importance should be given to the coordination between natural and artificial restoration measures to enhance ecosystems' resilience and reduce the dependence on human intervention.

The effectiveness of ecological programs depends on the combination of restoration measures and local socio-ecological systems (Chen and Cao, 2013; Petursdottir et al., 2013). The durability of these restoration interventions is heavily reliant on the delicate balance between restoration/protection efforts and residents' livelihood. If the implementation of these measures affects the residents' livelihood and their economic losses are not covered by ecological compensation, the residents will re-exploit the resources. In addition, the urban/agricultural expansion driven by increasing population and consumption is competing for the restored land (Wang et al., 2021). It is essential to diversify and maintain herders' revenue sources to overcome this. The paradigm of Sanjiangyuan National Park can be regarded as a viable choice. National parks can offer herders salaried work to participate in conservation programs or tourism, achieving a win-win situation since they increase their revenue and safeguard grasslands protection. This can also involve experienced indigenous people in the management of PAs, which international experience values (e.g., Garnett et al., 2018; Gonçalves-Souza et al., 2021). Also, ecological migration can be a solution, but this option requires appropriate production means and sufficient infrastructure. Sustainable grazing management merits additional investigation as well. In general, rotational grazing with appropriate grazing intensity (according to the grassland biogeographical characteristics), in the warm season, may preserve or improve soil fertility, species diversity, and overall ecosystem stability (Zhou et al., 2017; Sun et al., 2018; Dong et al., 2020). Furthermore, indefinite government payments are a potential source of funding through the substantial market of the ecosystem services provided by restoration interventions, such as the carbon market (Wang et al., 2021). Demand-side solutions to increase pasture productivity can also be considered. The current grazing model relies on a single herdsman family as the basic unit, increasing grazing fragmentation and hindering large-scale grazing production. With the urbanization process, the number of herds decreases, and grazing production will likely be dominated by large-scale livestock farming. This trend was already observed in many high-income countries. As a critical approach to agricultural modernization and sustainable development, large-scale grazing may positively impact herders' net profit and economic, technical, and labour efficiency and protect the environment through intensive production (Costs to the environment). This will maximize the efficiency of grassland use from the supply side and the allocation of land and labour resources.

In sum, grassland restoration must be approached from a socio-ecological system perspective. On the one hand, attention must be paid to the bidirectional constraints of residents' wellbeing (e.g., income, job opportunities) and environmental carrying capacity (e.g., water resource consumption). On the other hand, the implementation of grassland restoration should combine natural restoration with artificial restoration to ensure long-term effectiveness. Furthermore, improving the utilization efficiency of grassland resources can also alleviate the conflicts between people and nature to a certain extent. Diverse ecological protection and construction schemes on the QTP reversed the grassland degradation and conserved and restored natural capital. It may also increase economic and climatic resilience, enhance food security, and maintain biodiversity (FAO, 2021). The QTP's experience in grassland restoration can help other regions to contribute to humanity's shared sustainability pathway embodied in the SDGs.

## Author Contributions

TH and WZ conceived the study. TH and PP drew the graphs and wrote the manuscript. All authors reviewed and revised the manuscript. All authors contributed to the article and approved the submitted version.

## Conflict of Interest

The authors declare that the research was conducted in the absence of any commercial or financial relationships that could be construed as a potential conflict of interest.

## Publisher's Note

All claims expressed in this article are solely those of the authors and do not necessarily represent those of their affiliated organizations, or those of the publisher, the editors and the reviewers. Any product that may be evaluated in this article, or claim that may be made by its manufacturer, is not guaranteed or endorsed by the publisher.
